# Supplementary material for: Tissue-specific mouse mRNA isoform networks
Source: Sci Rep. 2019 Sep 27;9:13949. doi: 10.1038/s41598-019-50119-x (PMC6765046; doi:10.1038/s41598-019-50119-x)
Supplement: Supplementary file 1 — Supplementary Figures and Tables [file 41598_2019_50119_MOESM1_ESM.pdf]

Tissue-specific mouse mRNA isoform networks

Gaurav Kandoi, Julie A. Dickerson*
Bioinformatics and Computational Biology Program, Iowa State University, Ames, IA, USA
Department of Electrical and Computer Engineering, Iowa State University, Ames, IA, USA

*Corresponding author
E-mail: julied@iastate.edu (JAD)

Fig S1. Optimization of number of trees in random forest. A bar plot of performance metrics computed at different number of trees used in random forest. There is very little improvement in the performance beyond 100 trees, therefore, we have used 100 trees while developing TENSION. Abbreviations - ROC: Receiver Operating Characteristic; MCC: Matthews Correlation Coefficient.

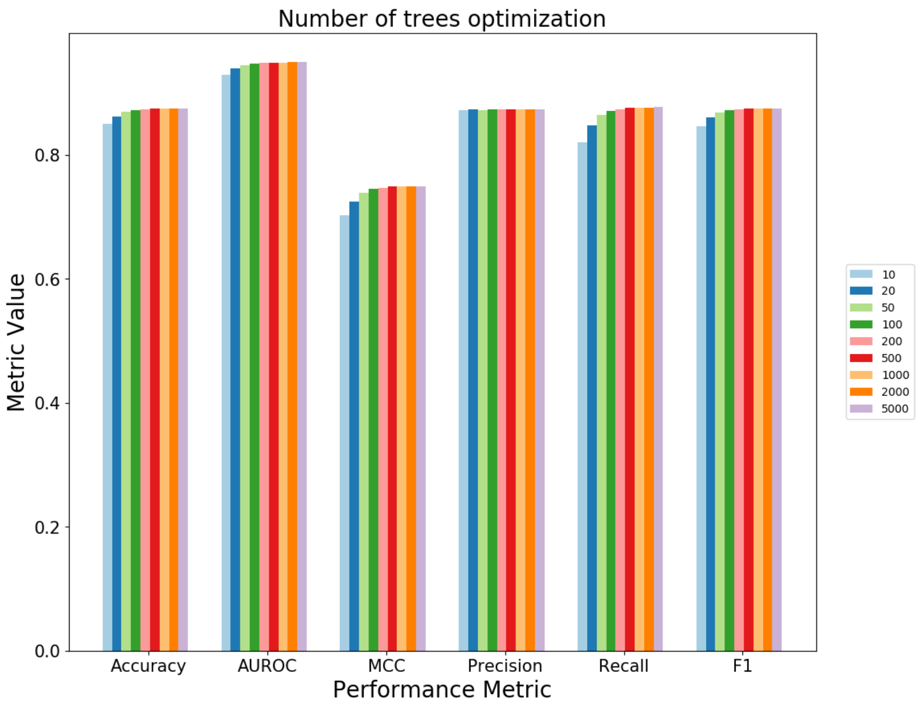

Fig S2. Performance evaluation on original testing dataset. The precision-recall and receiver operating characteristic curve for predictions on the original testing dataset. A model with area under the curve closer to 1 is better while a model with an area under the curve of 0.5 is equivalent to making random guess. Abbreviations - PR: Precision-Recall; ROC: Receiver Operating Characteristic.

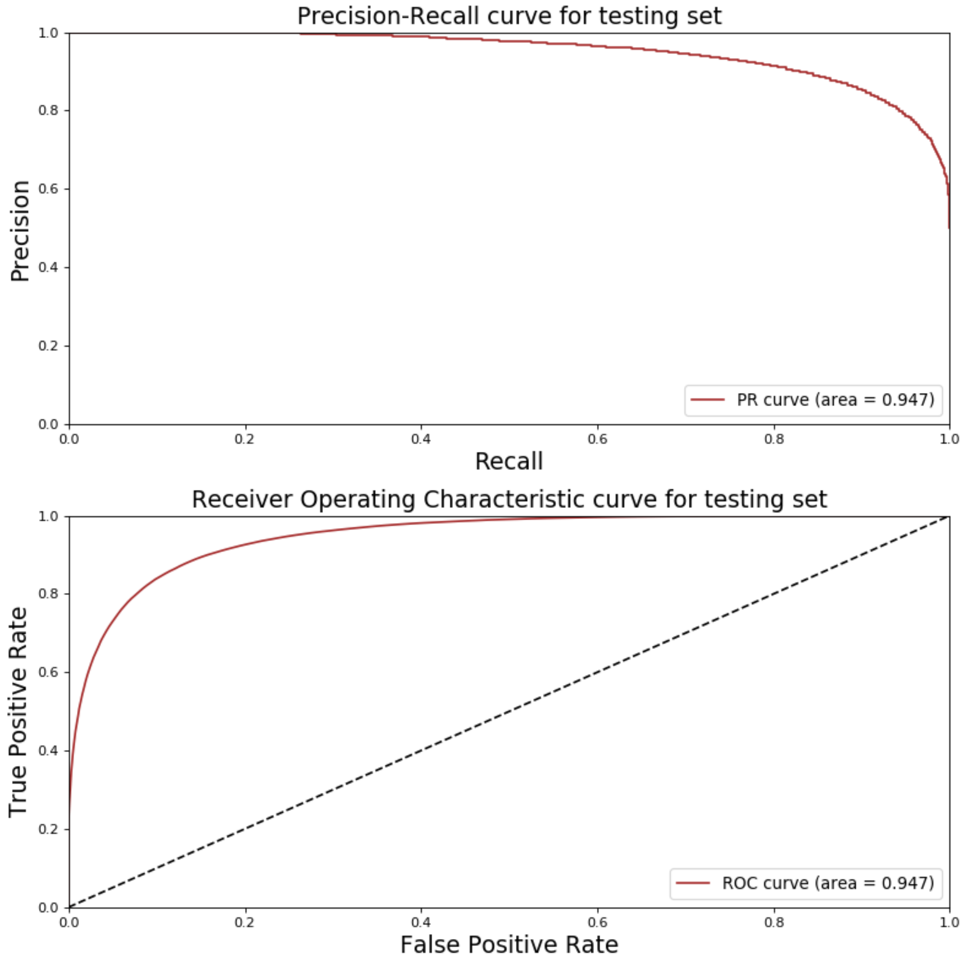

Fig S3. Evaluation of feature importance. The importance of all 27 features used to build TENSION was evaluated using the Gini index from Scikit-learn Random Forest. We see that some features are more than important than others. However, using all 27 features resulted in the best performance. Abbreviations – CTD: Conjoint Triad Descriptors; All: All RNA-Seq samples; Lintestine: Large intestine; AA: Amino Acid composition; DAC: Diamino Acid Composition; PAAC: Pseudo Amino Acid Composition; Moran: Moran Autocorrelation; AdGland: Adrenal glands; Sintestine: Small intestine; EmbFacPro: Embryonic facial prominence; Ntube: Neural Tube.

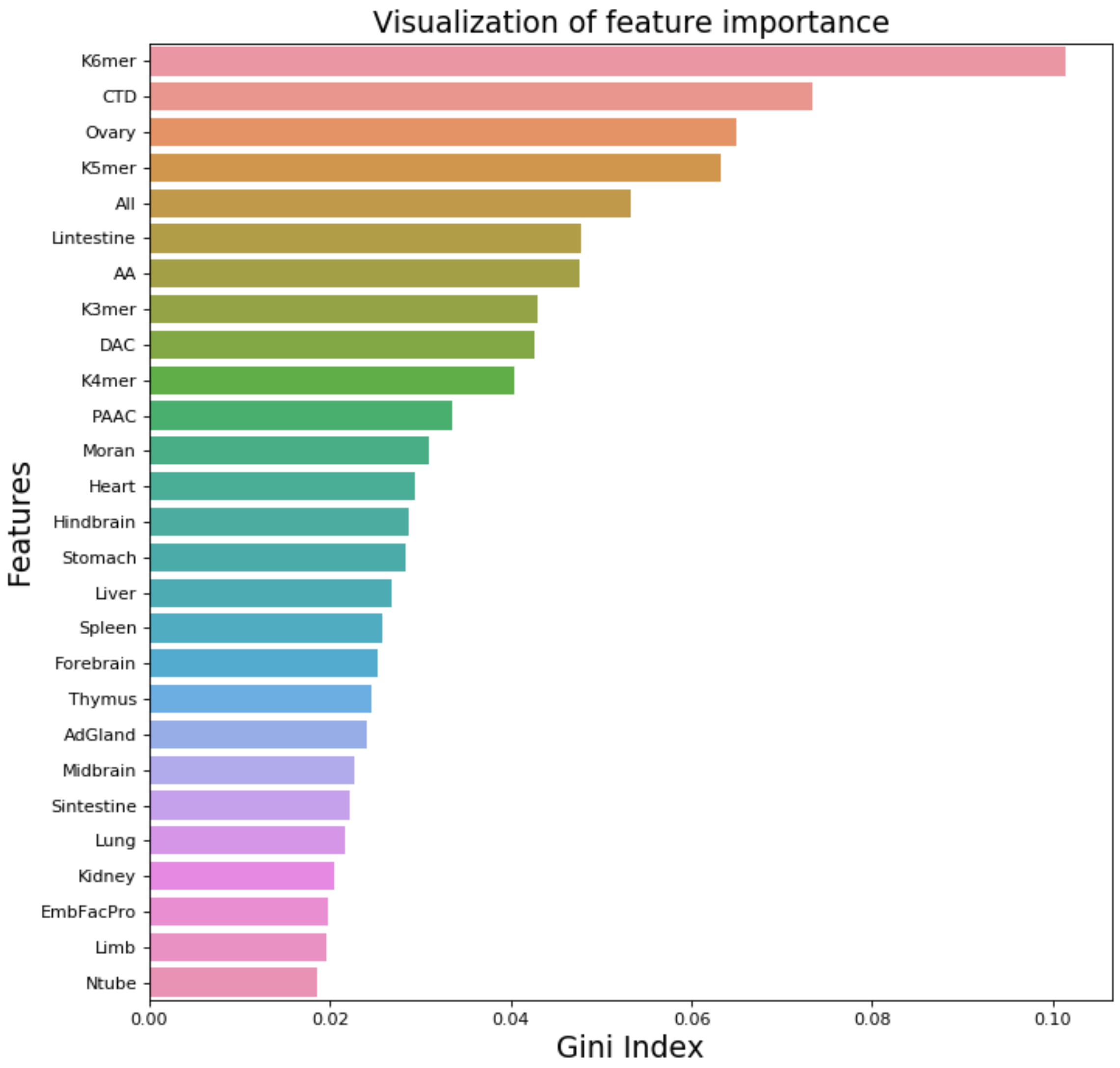

Fig S4. Fraction of mRNA isoform pairs functional in multiple tissues. The heatmap represents the fraction of mRNA isoforms pairs that are functional in two tissues. The numbers shown in the heatmap are not symmetric because the fraction is weighted by total mRNA isoforms pairs in that row's tissue. The fraction is weighted by the total number of pairs in the tissue specified on row. For instance, ovary shares 36.2% of mRNA isoform pairs present in the ovary network with large intestine. Darker shades refer to higher fractions of shared mRNA isoform pairs. The numbers in the heatmap should be interpreted as reading a matrix rowwise. Abbreviations - AdGland: Adrenal glands; EmbFacPro: Embryonic facial prominence; Ntube: Neural Tube; Sintestine: Small intestine; Lintestine: Large intestine.

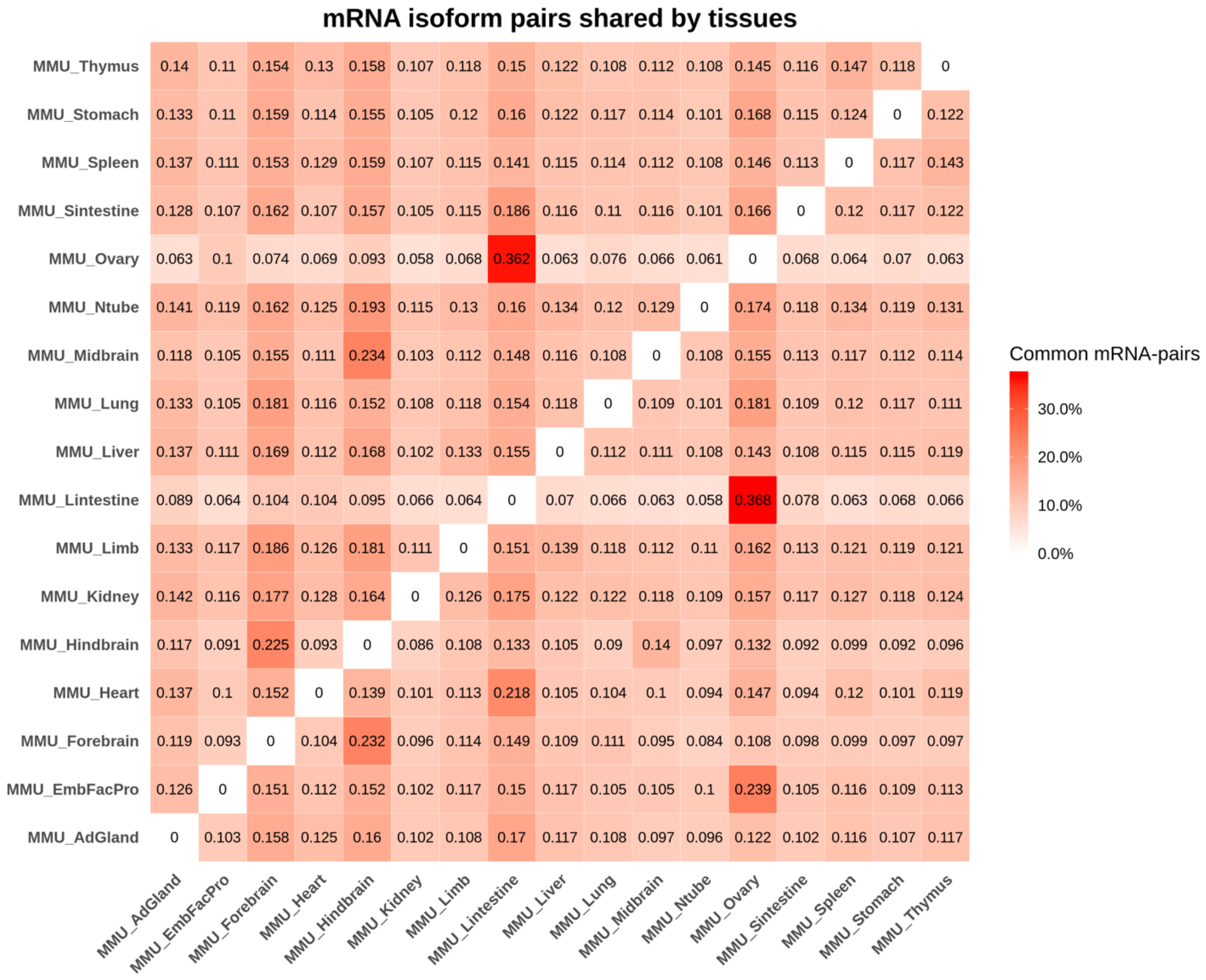

Table S1. List of RNA-Seq experiments and their tissue.

Sample	Tissue Type
ENCFF016KLR	adrenal gland
ENCFF360XMZ	adrenal gland
ENCFF694UNH	adrenal gland
ENCFF867HND	adrenal gland
SRR5047957	adrenal gland
SRR5047958	adrenal gland
SRR5047959	adrenal gland
SRR5047960	adrenal gland
SRR5047961	adrenal gland
SRR5047962	adrenal gland
SRR5048019	brain
SRR5048020	brain
SRR5048015	central nervous system
SRR5048016	central nervous system
SRR5048023	central nervous system
SRR5048024	central nervous system
SRR5048027	central nervous system
SRR5048028	central nervous system
SRR5048025	hindbrain
SRR5048026	hindbrain
SRR5047913	large intestine
SRR5047914	large intestine
SRR5047915	large intestine
SRR5047916	large intestine
SRR5047917	large intestine
SRR5047918	large intestine
SRR5048041	forebrain
SRR5048042	forebrain
ENCFF053CRD	embryonic facial prominence
ENCFF061AVT	embryonic facial prominence
ENCFF249ZZI	embryonic facial prominence
ENCFF252QAP	embryonic facial prominence
ENCFF316SZZ	embryonic facial prominence
ENCFF360GSG	embryonic facial prominence
ENCFF427WPK	embryonic facial prominence

ENCFF500GLW	embryonic facial prominence
ENCFF528UUE	embryonic facial prominence
ENCFF551UCM	embryonic facial prominence
ENCFF557LMN	embryonic facial prominence
ENCFF576MIX	embryonic facial prominence
ENCFF599OTY	embryonic facial prominence
ENCFF709FPA	embryonic facial prominence
ENCFF714ZDW	embryonic facial prominence
ENCFF744XBD	embryonic facial prominence
ENCFF771GDS	embryonic facial prominence
ENCFF781WVF	embryonic facial prominence
ENCFF839MMS	embryonic facial prominence
ENCFF839UKS	embryonic facial prominence
ENCFF896QPV	embryonic facial prominence
ENCFF917VEZ	embryonic facial prominence
ENCFF037JQC	forebrain
ENCFF114DRT	forebrain
ENCFF126IRS	forebrain
ENCFF179JEC	forebrain
ENCFF203BWA	forebrain
ENCFF235DNM	forebrain
ENCFF251LNG	forebrain
ENCFF270GKY	forebrain
ENCFF294JRP	forebrain
ENCFF320FJX	forebrain
ENCFF329ACL	forebrain
ENCFF358MFI	forebrain
ENCFF447EXU	forebrain
ENCFF458NWF	forebrain
ENCFF460TCF	forebrain
ENCFF528EVC	forebrain
ENCFF663SNC	forebrain
ENCFF700OLU	forebrain
ENCFF748SRJ	forebrain
ENCFF891HIX	forebrain
ENCFF896COV	forebrain
ENCFF920CNZ	forebrain

ENCFF920QAY	forebrain
ENCFF931IVO	forebrain
ENCFF959PSX	forebrain
SRR3192667	forebrain
SRR3192668	forebrain
SRR5047970	gonadal fat pad
SRR5047971	gonadal fat pad
SRR5047972	gonadal fat pad
SRR5047973	gonadal fat pad
ENCFF007SHF	heart
ENCFF019VGV	heart
ENCFF034XQS	heart
ENCFF063CEM	heart
ENCFF070OPW	heart
ENCFF136TII	heart
ENCFF220DJN	heart
ENCFF228OAV	heart
ENCFF229IFK	heart
ENCFF236BUE	heart
ENCFF358SEO	heart
ENCFF360NRX	heart
ENCFF381RLD	heart
ENCFF405TRT	heart
ENCFF418MSC	heart
ENCFF445AIZ	heart
ENCFF477PIC	heart
ENCFF478ZKL	heart
ENCFF485OXT	heart
ENCFF490QEC	heart
ENCFF500NCE	heart
ENCFF503TOH	heart
ENCFF586VFP	heart
ENCFF637ZYL	heart
ENCFF646JUX	heart
ENCFF676BDY	heart
ENCFF676PFC	heart
ENCFF878OGG	heart

SRR5047921	heart
SRR5047922	heart
SRR5047923	heart
SRR5047924	heart
ENCFF032XDZ	hindbrain
ENCFF060NTC	hindbrain
ENCFF094ZGK	hindbrain
ENCFF104CQE	hindbrain
ENCFF160LUK	hindbrain
ENCFF167NXN	hindbrain
ENCFF172XPK	hindbrain
ENCFF275SGM	hindbrain
ENCFF282QML	hindbrain
ENCFF336BOI	hindbrain
ENCFF372TIL	hindbrain
ENCFF377BWR	hindbrain
ENCFF378HXV	hindbrain
ENCFF416EWW	hindbrain
ENCFF548GUA	hindbrain
ENCFF645LDN	hindbrain
ENCFF672PAZ	hindbrain
ENCFF700EWM	hindbrain
ENCFF706XOL	hindbrain
ENCFF738FUB	hindbrain
ENCFF786VDJ	hindbrain
ENCFF863UFD	hindbrain
ENCFF874AXO	hindbrain
ENCFF876NSY	hindbrain
ENCFF913XMQ	hindbrain
ENCFF926BFE	hindbrain
SRR3192647	hindbrain
SRR3192648	hindbrain
ENCFF014ZBI	intestine
ENCFF039KJW	intestine
ENCFF093ZAR	intestine
ENCFF107WVN	intestine
ENCFF113UJZ	intestine

ENCFF235BLS	intestine
ENCFF316QLU	intestine
ENCFF379JZS	intestine
ENCFF499WIP	intestine
ENCFF553BVK	intestine
ENCFF758VQQ	intestine
ENCFF904JAW	intestine
ENCFF021BPG	kidney
ENCFF070BUP	kidney
ENCFF140MLD	kidney
ENCFF143NRY	kidney
ENCFF266SYA	kidney
ENCFF301QEB	kidney
ENCFF367OPA	kidney
ENCFF652UDJ	kidney
ENCFF654QAE	kidney
ENCFF798JOI	kidney
ENCFF901TLF	kidney
ENCFF929PSZ	kidney
SRR5047925	kidney
SRR5047926	kidney
SRR5047927	kidney
SRR5047928	kidney
SRR5047929	kidney
SRR5047930	kidney
SRR5047975	large intestine
SRR5047976	large intestine
SRR5047977	large intestine
SRR5047978	large intestine
ENCFF184ELK	limb
ENCFF235PJS	limb
ENCFF237DCF	limb
ENCFF237SXT	limb
ENCFF246JLP	limb
ENCFF249AZE	limb
ENCFF262CIY	limb
ENCFF291NWK	limb

ENCFF409ZNA	limb
ENCFF419QRX	limb
ENCFF479HKB	limb
ENCFF565KTC	limb
ENCFF654OBR	limb
ENCFF678XFK	limb
ENCFF679RDZ	limb
ENCFF682WAX	limb
ENCFF775HDI	limb
ENCFF780HRS	limb
ENCFF820NAK	limb
ENCFF959ZAX	limb
SRR5048029	limb
SRR5048030	limb
ENCFF085URY	liver
ENCFF130DKL	liver
ENCFF155LJD	liver
ENCFF245OTN	liver
ENCFF276ENR	liver
ENCFF377KCE	liver
ENCFF473WMT	liver
ENCFF510RXX	liver
ENCFF526QHV	liver
ENCFF528MAS	liver
ENCFF536HIT	liver
ENCFF584CMS	liver
ENCFF635VBU	liver
ENCFF635YMK	liver
ENCFF677ULG	liver
ENCFF746XUK	liver
ENCFF810MMJ	liver
ENCFF854WTE	liver
ENCFF932YNB	liver
ENCFF956HCY	liver
ENCFF985XAR	liver
ENCFF986WFE	liver
SRR3192469	liver

SRR3192470	liver
SRR5047931	liver
SRR5047932	liver
SRR5047933	liver
SRR5047934	liver
SRR5047935	liver
SRR5047936	liver
SRR5048017	liver
SRR5048018	liver
SRR5048021	liver
SRR5048022	liver
SRR5048031	liver
SRR5048032	liver
ENCFF289EZB	lung
ENCFF503BOB	lung
ENCFF618MYZ	lung
ENCFF657LQI	lung
ENCFF728LAM	lung
ENCFF778KZE	lung
ENCFF800SJE	lung
ENCFF910RNP	lung
ENCFF916XIQ	lung
ENCFF919VVI	lung
SRR5047937	lung
SRR5047938	lung
SRR5047939	lung
SRR5047940	lung
ENCFF051VXS	midbrain
ENCFF052VGB	midbrain
ENCFF059FUK	midbrain
ENCFF062HAD	midbrain
ENCFF091YUW	midbrain
ENCFF093YSD	midbrain
ENCFF099UUS	midbrain
ENCFF156BSL	midbrain
ENCFF327YJQ	midbrain
ENCFF348BYM	midbrain

ENCFF421QJA	midbrain
ENCFF476UXE	midbrain
ENCFF499UQZ	midbrain
ENCFF727ACE	midbrain
ENCFF739QUZ	midbrain
ENCFF810ZDM	midbrain
ENCFF819IDW	midbrain
ENCFF819ZTA	midbrain
ENCFF839VKV	midbrain
ENCFF853SOX	midbrain
ENCFF889DNO	midbrain
ENCFF938RVG	midbrain
SRR3192588	midbrain
SRR3192589	midbrain
ENCFF003CSR	neural tube
ENCFF046EJC	neural tube
ENCFF064MCV	neural tube
ENCFF078SPI	neural tube
ENCFF085MBO	neural tube
ENCFF090KDU	neural tube
ENCFF198HBZ	neural tube
ENCFF216XBD	neural tube
ENCFF241GLU	neural tube
ENCFF321ZGR	neural tube
ENCFF378CNA	neural tube
ENCFF405VKS	neural tube
ENCFF447CLP	neural tube
ENCFF489RVW	neural tube
ENCFF528JFG	neural tube
ENCFF555MUK	neural tube
ENCFF581SPK	neural tube
ENCFF739BEA	neural tube
ENCFF758NAG	neural tube
ENCFF834WRE	neural tube
ENCFF895DPO	neural tube
ENCFF957SPL	neural tube
SRR5047985	ovary

SRR5047986	ovary
SRR5047987	ovary
SRR5047988	ovary
SRR5047989	ovary
SRR5047990	ovary
SRR5047991	ovary
SRR5047992	ovary
SRR5047993	ovary
SRR5047994	ovary
SRR5171100	ovary
ENCFF300QQW	skeletal muscle tissue
ENCFF494GEO	skeletal muscle tissue
ENCFF562GFS	skeletal muscle tissue
ENCFF642EVR	skeletal muscle tissue
SRR5048001	small intestine
SRR5048002	small intestine
SRR5048003	small intestine
SRR5048004	small intestine
SRR5048005	small intestine
SRR5048006	small intestine
SRR5048007	small intestine
SRR5048008	small intestine
SRR5048009	small intestine
SRR5048010	small intestine
SRR5171080	small intestine
ENCFF014ITK	spleen
ENCFF063LXW	spleen
ENCFF082DGE	spleen
ENCFF671GFL	spleen
SRR5047941	spleen
SRR5047942	spleen
SRR5047943	spleen
SRR5047944	spleen
SRR5047945	spleen
SRR5047946	spleen
ENCFF180LOF	stomach
ENCFF263TIR	stomach

ENCFF273IHW	stomach
ENCFF352EXD	stomach
ENCFF417NAF	stomach
ENCFF494GQK	stomach
ENCFF553BSP	stomach
ENCFF775CBB	stomach
ENCFF850KIG	stomach
SRR5047995	stomach
SRR5047996	stomach
SRR5047997	stomach
SRR5047998	stomach
SRR5047999	stomach
SRR5048000	stomach
SRR5047953	testis
SRR5047954	testis
SRR5047955	testis
SRR5047956	testis
ENCFF453JXA	thymus
ENCFF530SXN	thymus
ENCFF638TAT	thymus
ENCFF718DNJ	thymus
SRR5047947	thymus
SRR5047948	thymus
SRR5047949	thymus
SRR5047950	thymus
SRR5047951	thymus
SRR5047952	thymus
SRR5048035	urinary bladder
